# The unfolded protein response in glioblastomas: targetable or trouble?

**DOI:** 10.4155/fso.15.45

**Published:** 2015-09-01

**Authors:** Michael W Graner

**Affiliations:** 1 University of Colorado Denver (Anschutz Medical Campus), Aurora, CO 80045, USA

**Keywords:** brain tumor, chaperone, clinical trial, glioblastoma, glucose-regulated proteins, heat shock proteins, HSP90 inhibitors

## Abstract

Glioblastomas are devastating central nervous system tumors with abysmal prognoses. These tumors are often difficult to resect surgically, are highly invasive and proliferative, and are resistant to virtually all therapeutic attempts, making them universally lethal diseases. One key enabling feature of their tumor biology is the engagement of the unfolded protein response (UPR), a stress response originating in the endoplasmic reticulum (ER) designed to handle the pathologies of aggregating malfolded proteins in that organelle. Glioblastomas and other tumors have co-opted this stress response to allow their continued uncontrolled growth by enhanced protein production (maintained by chaperone-assisted protein folding) and lipid biosynthesis driven downstream of the UPR. These features can account for the extensive extracellular remodeling/invasiveness/angiogenesis and proliferative capacity, and ultimately result in tumor phenotypes of chemo- and radio-resistance. The UPR in general, and its chaperoning capacity in particular, are thus putative high-value targets for treatment intervention. Such therapeutic strategies, and potential problems with them, will be discussed and analyzed.

**Figure 1.**
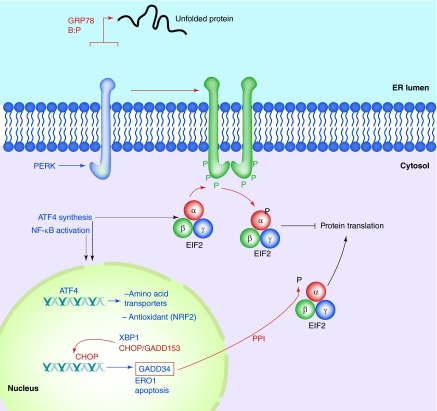
**PERK in the unfolded protein response.** As unfolded proteins appear in the ER lumen, the chaperone GRP78 releases PERK, allowing the latter to dimerize in the ER membrane, resulting in autophosphorylation. This activates PERK's kinase domain, phosphorylating eIF2α and halting translation in the cytosol. *ATF4* mRNA is preferentially translated, and that transcription factor drives gene expression of amino acid transporters, anti-oxidant genes, XBP-1, CHOP/GADD153 and GADD34. CHOP may lead to apoptotic induction, while GADD34 is a subunit of the type 1 protein ser/thr phosphatase PPI, which dephosphorylates eIF2α to resume translation.

**Figure 2.**
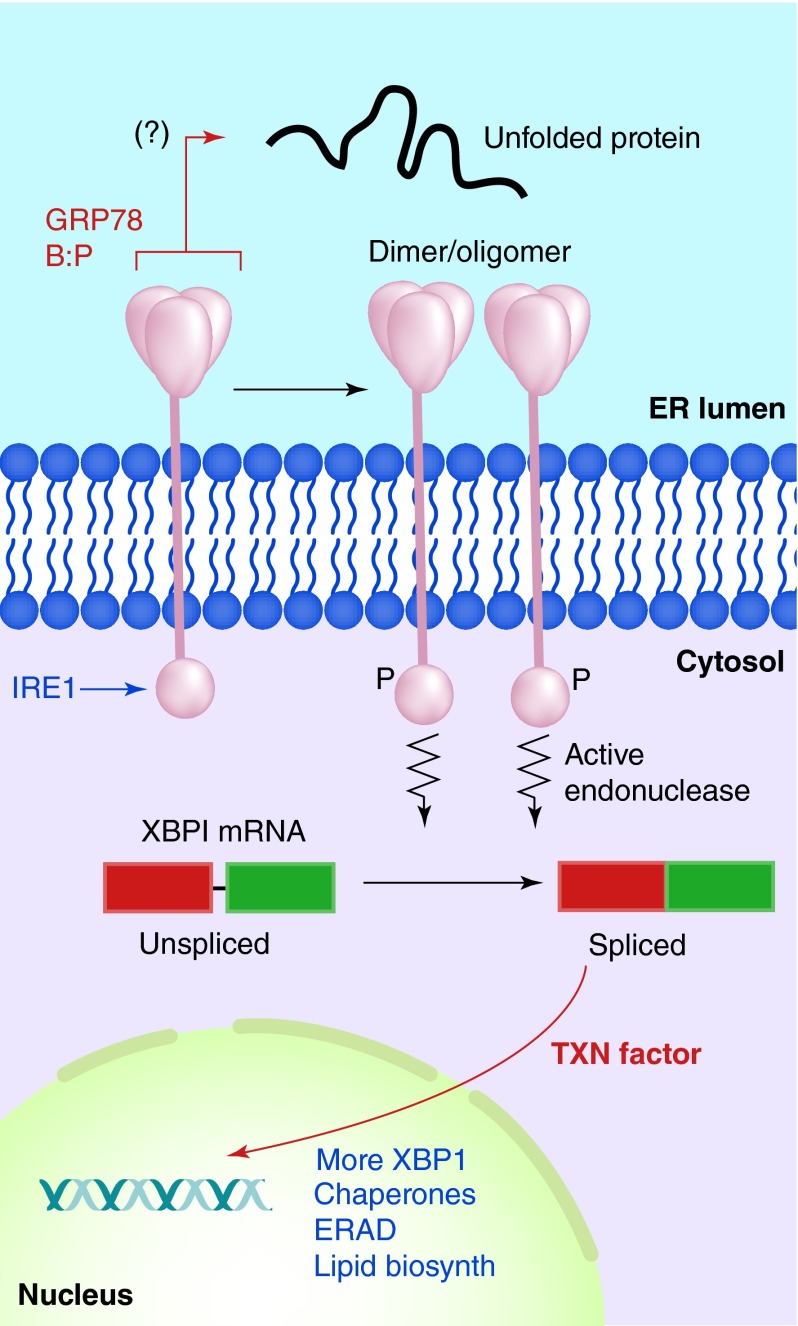
**IRE1 in the unfolded protein response.** Accumulation of unfolded proteins in the ER lumen is sensed by the IRE1 lumenal domain (with possible release from GRP78), leading to dimerization or oligomerization. This results in autophosphorylation and kinase activation in IRE1's cytosolic domains, which induces IRE1 ribonuclease activity, allowing for the splicing of *XBP-1* mRNA. The new stable transcription factor drives gene transcription for increased chaperone output, lipid biosynthetic enzymes, components of the ERAD degradation system and more XBP-1.

**Figure 3.**
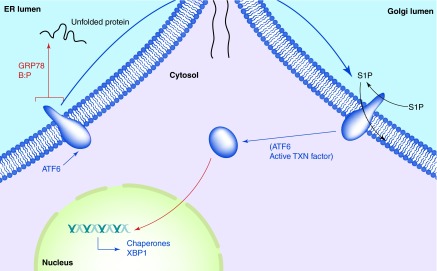
**ATF6 in the unfolded protein response.** Accumulation of unfolded proteins in the ER lumen leads to the release of GRP78 from the lumenal domain of ATF6, which can now traverse into the Golgi. There, Golgi resident proteases S1P and S2P cleave ATF6's intramembrane region, releasing the active transcription factor into the cytosol and nucleus. Among the genes activated by ATF6 are those encoding chaperones and yet more XBP-1.

**Figure 4.**
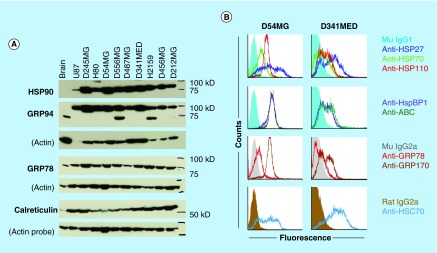
**Brain tumors overexpress chaperone proteins and may localize them to the cell surface.** **(A)** Western blots of lysates from xenografts of various adult and pediatric gliomas and a medulloblastoma were probed for HSP90, GRPs 94 and 78, and calreticulin. Normal rat brain is used as a comparator tissue, and probes for actin are shown as loading controls. **(B)** FACS analyses of GBM (D54MG) and medulloblastoma (D341MED) cell lines with surface staining for heat shock and chaperone proteins shown (HspBP1 is an HSP70 family co-chaperone; ABC is α-B crystallin). Background from murine or rat isotype control antibodies is shown in dark fill.

**Figure 5.**
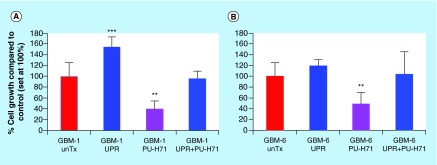
**Glioblastoma ‘stem cell’ lines following UPR induction resist treatment with an HSP90 inhibitor.** Primary cell lines (**[A]** GBM-1, from a recurrent tumor, and **[B]** GBM-6, from a primary tumor) were generated in stem cell medium and were cultured as ‘neurospheres’. Cells were treated (or not) with dithiothreitol to induce the unfolded protein response. Groups of cells were then treated (or not) with very high doses of the HSP90 inhibitor PU-H71 (100 mM) for 48 h. Cell proliferation was measured by MTS assay and is set to 100% for untreated controls (red bars). Statistical differences in proliferation were determined by *t*-test compared to untreated controls. **p < 0.01; ***p < 0.005.

**Figure 6.**
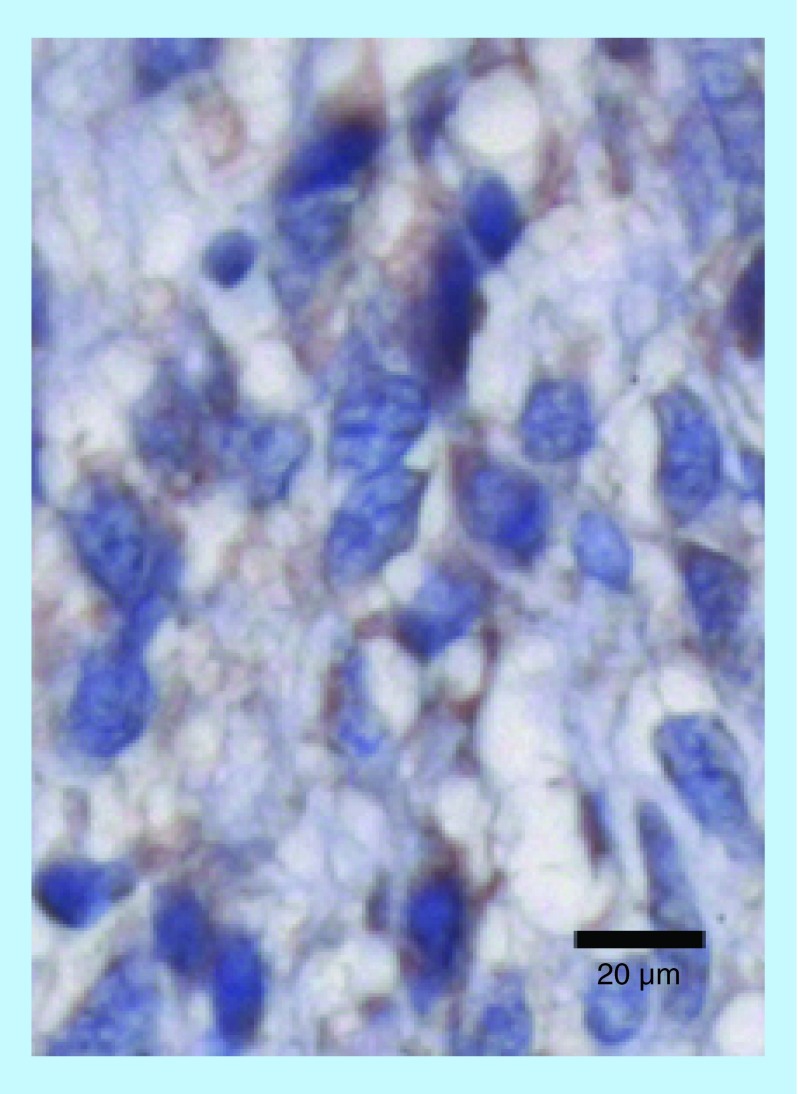
**The proapoptotic transcription factor CHOP/GADD153 may be sequestered in the cytosol in some brain tumors.** CHOP may be induced by the PERK arm of the UPR (Figure 1). Shown is an immunohistochemical stain for CHOP on 5 μm paraffin sections of a D245MG GBM xenograft tumor (IHC details are in Epple *et al*. [12]). Counterstain is hematoxylin/eosin (resulting in blue nuclear staining, while brown staining is for the CHOP protein). Nearly all of the nuclei are spared of the immunostain, suggesting that CHOP cannot enter the nuclei.

Glioblastoma multiforme (WHO grade IV glioma [GBM]) is a devastating tumor of the brain/central nervous system, and is also the most common malignant primary brain tumor in the USA. Patients with GBMs seldom live longer than 15 months [1], and this number has barely changed in the past two decades [2]. The continual damage to the brain from the tumor itself as well as from our current therapies leads to debilitating physical, neurocognitive and psychological sequelae while the patient is still alive. Clearly, our existing treatment regimens are unacceptable, and we need a far greater understanding of the biology of these tumors to address improved therapeutics.

## Tumor stressors & the unfolded protein response

Solid tumors are existentially stressed tissues. Their disregard for normalizing cues concerning unrestrained cell proliferation leads to inadequate blood supplies, insufficient nutrients and a hypoxic environment. The host's immune system, while largely inadequate, nonetheless attacks the tumor and requires resistance, and exogenously we attempt to inflict damage with chemical agents and radiation. These stresses provoke tumors to defend themselves by upregulation of particular pathways designed for cytoprotection, particularly those involved in protein folding and protein stabilization. These pathways include upregulation of chaperone proteins/heat shock proteins in the cytoplasm (and other organelles such as the mitochondria and endoplasmic reticulum), including those involved in the unfolded protein response (UPR) [3,4]. GBMs follow these patterns, as well [5–7].

Ostensibly, the UPR manifests when misfolded proteins accumulate in the endoplasmic reticulum (ER) [6], but the effects extend beyond that organelle and ultimately involve the entire cell. This multifaceted phenomenon classically follows from stresses that might lead to malfolded or unfolded proteins under conditions typically present in tumors: loss of redox control [7], possibly due to overburdening of the ER with excessive protein during rampant cell proliferation; loss of oxidative capacity in hypoxia [8]; or glucose starvation leading to metabolic disarray and a lack of substrate sugars for protein glycosylation [9]. Tumors will invoke the UPR to counteract the detrimental environmental effects (often of the tumor's own making) [10]. Ultimately, the benefits of the stress response outweigh the risks (e.g., apoptosis) [11] and the tumor's engagement of the UPR becomes ‘fixed’ [12]. This is logical in a cellular situation where there is need for dynamic cell surface and extracellular/microenvironmental remodeling [13,14] to promote angiogenesis or tumor cell migration/invasion/metastasis. Such cell surface and extracellular reorganizations require an active secretory pathway that is evident in tumor cells [15], and the UPR contributes to that.

## The UPR & its sensors, transducers & effectors

The UPR is a beautifully complex set of molecular interactions that globally alter cellular transcriptional and translational profiles, involving sensors, transducers and multiple layers of effectors. One of the ER sensors is the HSP70 family member GRP78 (BiP/HSPA5); in its chaperone role it can detect unfolded/malfolded proteins in the ER lumen, and can assist in their folding in an ATP-dependent manner [16]. Under conditions of cellular stasis, GRP78 is known to bind to the ER-lumenal portions of three transmembrane molecules: PERK, IRE1, and ATF6 [17].

With GRP78 bound to these three ER membrane proteins, they remain in a monomeric/tethered state; once some triggering level of unfolded proteins appears in the ER lumen, GRP78 releases the three to engage in chaperone duties for the unfolded proteins. PERK and IRE1 now can dimerize or oligomerize, and ATF6 simply leaves the organelle, to act as transducers of the UPR (IRE1 has its own unfolded protein sensing domain [6] and may not require GRP78's tethering services, although GRP78 may modify IRE1's activities). PERK and IRE1 dimerize or form higher-order oligomers with autokinase activities, while ATF6 is released into the Golgi for proteolytic processing (Figures 1–3).

PERK oligomerizes by its N-terminal ER-lumenal domain via two regions, one of which binds GRP78 (GRP94 also binds the N-terminal portion of PERK, but the binding site is unclear [18]). The dimerization/oligomerization leads to transautophosphorylation of the C-terminal, cytoplasmic domain. Multiple sites are phosphorylated, including sites in the kinase activation loop [19]. It is this kinase activation loop that phosphorylates eIF2α/EIF2A. Phospho-eIF2α inhibits translational initiation, thus bringing protein translation to a halt, which reduces the burden of unfolded proteins amassing in the ER.

Phosphorylation of eIF2α inhibits eIF2B (a guanine exchange factor), reducing the GDP-GTP exchange of eIF2-GTP. This prevents binding of eIF2 to initiator methionine tRNAs and loading into the P site on the 40S ribosome, effectively halting most translation [20]. However, certain mRNAs such as that of *ATF4* are translated more efficiently in this situation (the *ATF4* 5′ UTR has several short open reading frames that are skipped as the 40S ribosome scans past them to find the appropriate start site [21]). In addition, reduced translation results in loss of IκB due to degradative turnover, which allows entry of NFκB into the nucleus to activate transcriptional programs associated with it [22], such as inflammatory mediators [23]. Some of the downstream consequences of ATF4 activation include transcriptional increases in amino acid transporters and antioxidant signaling (e.g., NRF2, and phospho-PERK directly phosphorylates NRF2 to activate it [24]), XBP-1 (see below), CHOP/GADD153 and GADD34 (which is also a transcriptional target of CHOP). The relationship between ATF4, CHOP and GADD34 is interesting in that GADD34 is a subunit of PPI (a serine/threonine phosphatase) which dephosphorylates eIF2α, releasing the PERK-driven translational block of protein synthesis [25]. However, CHOP is a transcription factor tied to downstream apoptotic players potentially leading to cell death [26]; thus, the interplay between the re-engaging of protein synthesis and the activation of apoptosis can be seen as a ‘hedging of bets’, setting the cell up for recovery or demise depending on the extent of the stress and input from other signals (Figure 1).

IRE1 dimerization/oligomerization of its N-terminal lumenal domain results in transautophosphorylation and kinase domain activation in its C-terminal cytoplasmic domain. A conformational change induces a unique endoribonuclease function which excises a 26 base intron from *XBP-1* mRNA (called the ‘unspliced’ form, *XBP-1u*), and allows splicing (by unknown ligases) to generate the longer (‘spliced’, *XBP-1s*) mRNA with a frame shift that now codes for a longer, more stable and active protein transcription factor. IRE1 is also responsible for degradation of other mRNAs, particularly those encoding membrane and secreted proteins [27], in a pathway termed regulated IRE1-dependent decay (RIDD) reducing the protein folding burden within the ER (although this is differentially regulated from the *XBP-1* cleavage). As a transcription factor, XBP-1 transcribes genes encoding for increased ER chaperone production (e.g., GRPs 78, 94 and 170, as well as members of the PDI family), and also, more XBP-1. In addition, there is upregulation of members of the ERAD pathways (Endoplasmic Reticulum Associated Degradation) and lipid biosynthetic enzymes. These various outcomes from IRE1 dimer-/oligomerization lead to improved folding of proteins (chaperones) and transport out of the ER (lipid synthesis) or increased clearance of unfoldable proteins (ERAD) and reduced translation of proteins entering the ER (RIDD), thus relieving the unfolded protein ER stress (Figure 2).

Upon stress-induced release of ATF6 from GRP78, the transmembrane protein traverses the ER and into the Golgi via COPII vesicles, where its intramembrane region is cleaved by proteases S1P and S2P [28]. The N-terminal cytosolic portion is now freed to enter the nucleus as an active transcription factor [29], where it activates perhaps approximately 300 genes [30], including those involved in chaperone and protein folding functions, as well as yet more XBP-1 (Figure 3).

These three arms of the UPR work interdependently to alleviate the energetic, metabolic and oxidative stress on the ER in the presence of accumulating unfolded proteins. At least some of the chaperone folding machinery utilizes ATP for its function. This is also done in the presence of calcium (the ER is the major calcium storage organelle in the cell), and requires significant inputs of amino acids, lipids and carbohydrates (as the ER is also the site of initial glycoprotein formation and vesicular packaging for transport to other organelles). As disulfide bonds are formed, oxidative equivalents are taken, and if the proteins are to be secreted, the entire ‘product’ can be viewed as being lost from the cellular pool of biochemicals. Thus, the UPR attempts to retain stasis in a finely-tuned system where stresses could prove catastrophic – leading to apoptotic induction if the stresses prove insurmountable.

## The unfolded protein response in glioblastomas

The UPR in GBMs has not been extensively studied, despite the indicators suggesting that the UPR should be activated in these cells: GBMs show rapid proliferation [31,32], which requires biosynthesis and mobilization of large quantities of lipids; GBMs are migratory and highly invasive, as well as angiogenic [33,34], thus necessitating an active extracellular secretory process and a dynamic cell surface microenvironment; and they are intrinsically or adaptively resistant to essentially all chemotherapeutics and can tolerate high external beam radiation [35–37] displaying elevated stress responses against internal and external offenses [38–40]. These devastating characteristics of GBMs are enabled by the UPR, driving both secretory pathway function and promoting stress resistance via altered metabolism [41–43].

Recently, our group characterized aspects of the UPR in established and primary GBM cell lines, in xenograft tumors, and in clinical surgical samples [12]. Among other things, we noted that in all GBM samples, many elements of the UPR were elevated particularly in the solid tumors, and that mRNA expression of GRPs 78 and 94, as well as XBP-1, correlated with worse prognosis. Additionally, GBM cells undergoing UPR stress initially halted protein production, but within an hour had resumed protein synthesis, climbing to near normal levels by 4 h – despite continuous culture in the presence of the stressor during that 4-h period. The return of protein synthesis eventually led to increased proliferation of GBM cells following UPR stress along with a profound resistance to chemotherapy. These features of the UPR and the recovery or ‘stasis’ of the cells with it, all suggest a highly active chaperoning/protein folding system within brain tumors.

## Chaperone proteins & brain tumors

We have covered this topic at length in previous publications [5,44–46], but some concepts deserve revisiting. One is that HSPs and other chaperones (GRPs 75, 78, 94, 170, calreticulin, the PDI family members, *etc*.) are all upregulated in brain tumor cells from a variety of sources (high grade gliomas, pediatric gliomas, medulloblastomas, ependymomas, *etc.*), and many are displayed on the surfaces of such tumor cells [5,12,46–48]. Figure 4A presents an example of Western blots for HSP90 and ER chaperones GRPs 94 and 78, and calreticulin across a number of brain tumor xenografts compared with normal rodent brain, where there are in some cases extraordinarily high expression levels of the chaperones in tumors. Figure 4B shows FACS analyses of chaperones/HSPs on the surfaces of glioma and medulloblastoma cells (with no such staining on the surfaces of disaggregated brain cells [5]). These expression levels imply functional utility driven by rapid proliferation and an accompanying need for properly folded proteins, along with the intrinsic capabilities of chaperones to inhibit cellular apoptosis by interactions with proapoptotic factors [49,50]. While it is not clear if this high expression and presumed functions of chaperones in brain tumors could be considered a form of ‘nononcogene addiction’, these phenomena do suggest that targeting chaperones could be a reasonable cancer therapeutic strategy [45,51]. Thus, the second point would be, can these proteins/activities be targeted?

We reviewed the pharmacologies of brain tumor chaperone protein inhibition before [44,45]; currently there are no clinical trials for patients with brain tumors that employ such inhibitors, and all of the inhibitors in clinical use target HSP90 [52]. While sometimes touted as potentially useful chemotherapies for gliomas, HSP90 inhibitors such as the geldanamycin derivative 17-AAG often are used at rather high concentrations to exert effects. For instance, 17-AAG IC_50_ values from 50 nM to nearly 500 nM were used when treating various glioma cell lines [53]. These values are 10- to 100-fold higher than for many other cancer cell lines [54,55]. Later generation HSP90 inhibitors of the SNX-2112 class [56] or the PU-H71 class [57] show tremendous *in vitro* effectiveness against a variety of tumor cells (1< nM to 300 nM), but in our hands did not have any such effects, despite a potential relationship between SNX-2112 HSP90 inhibition and the UPR [58] (however, see Figure 5, where the HSP90 inhibitor PU-H71 was used at 100 μM for 48-h treatment; other data not shown). PU-H71 also inhibits the HSP90 paralog GRP94 in multiple myeloma cells in a manner that actually induces the UPR [59]. However, against glioma stem cell lines, induction of the UPR completely abrogates the effect of the drug (Figure 5), just as U87MG cells resist temozolomide upon UPR induction [12]. While we suspect that enhanced metabolism following UPR stress may be a mechanism for this resistance [12], this awaits further study. Another inclusive option is that high overexpression and constant replenishment (i.e., continuous protein synthesis of chaperones) make chaperones difficult targets in chronically stressed brain tumor cells. Such results suggest that HSP90 inhibitors may not be suitable single agents against gliomas, and may be among the reasons that there are currently no HSP90 inhibitors in GBM clinical trials.

Are other ER chaperones viable targets in GBMs? Previous work has demonstrated a solid role for GRP78 as a potential chemosensitizer target in GBMs [60], where knockdown of the message or treatment with epigallocatechin gallate (binds to the nucleotide-binding domain necessary for chaperone/folding function) sensitized glioma cells to various chemotherapies, including temozolomide. However, overexpression of GRP78 led to increased resistance and enhanced cell viability, and this is a hallmark of the UPR both in that study and in our work (Figure 4) [12]. One ‘take-home message’ from the cumulative results of these aforementioned studies and data would be that researchers should perform drug treatment studies *in vitro* with UPR-stressed cells. That setting would perhaps more closely resemble the stresses cells face *in vivo* and give a more accurate assessment of pharmacologic efficacy of a particular agent.

## Other targets from the UPR?

There are potentially many targets within the cohort of sensors, transducers and effectors in the UPR, but there have been few successes in terms of cancer therapeutics. The kinase activity of PERK is a logical point of intervention, and rationally designed inhibitors are available (e.g., [61,62]) but none are in clinical trials. Our previous results might suggest that PERK does not play an integral role in the maintenance of the UPR in gliomas [12], since we found that eIF2α phosphorylation is transient in stressed GBM cells. We also noted that in recurrent GBMs, the ATF6 and IRE1 pathways seemed most engaged, and the IRE1/XBP-1 axis is a prevalent theme in the GBM UPR literature [63]. What few inhibitors of IRE1 exist target its endonuclease domain [64–67] rather than the kinase activity, since the conformational status of the protein rather than its kinase function is key for the endonuclease activity [68] (however, FDA-approved sunitinib, an inhibitor of several receptor tyrosine kinases, does appear to affect IRE1 [69], and has been used in numerous clinical trials for GBM patients). None of the cited IRE1 inhibitors appear to have been employed in a brain tumor setting thus far.

Downstream of both IRE1 and ATF6 is XBP-1. Direct inhibition of XBP-1 transcriptional activities has not been described. Alternatively, the triene-ansamycins were identified as compounds preventing the splicing of XBP-1 mRNA [70]. None of these compounds appear to have reached clinical trial stages as yet.

Conceptually, ATF6 might be inhibited directly, but no compounds currently seem to do this – however, such screening may be underway [71]. ATF6 could be targeted less directly by preventing its selective proteolysis by the S1P and S2P Golgi proteases [72]. Nelfinavir is one such protease inhibitor capable of affecting ATF6 processing [73] and is part of a proposed treatment approach for GBMs utilizing repurposed drugs [74]. There is one clinical trial listed for patients with GBMs using nelfinavir (NCT00915694) but its status is currently unknown.

In general, pharmacologic inhibition of transcription factors has historically been considered difficult, usually due to the lack of enzymatic activities associated with such proteins [75]. Thus, direct targeting of ATF6, ATF4 and XBP-1 may not be reasonable, and their pleiotropic downstream effects could make for complicated evaluations. However, attempts to increase expression and/or activities of some transcription factors might be viable options for apoptosis inducers such as CHOP/GADD153. One of the many effects of the isoflavone genistein is the upregulation of CHOP [76]. However, in high grade gliomas, we found CHOP expression to be highly variable [12], and not correlated to apoptotic induction. Indeed, the relationships between CHOP expression and downstream modifiers (dimerization partners, posttranslational modifications) are complex and may not necessarily lead to cellular apoptosis [77]. We have noticed that CHOP localization intracellularly may be a relevant factor as well. In Figure 6 we show by immunohistochemistry that CHOP is expressed in this GBM xenograft tumor (brown deposition), but the protein is not found in the nuclei (large blue bodies). One may speculate that the abundance of chaperones may play a role in sequestration of CHOP, but there is no evidence in the literature to support this.

## Targeting processes related to the UPR

As mentioned above (Figure 2), ERAD is invoked to remove and degrade unfolded or unfoldable proteins from the ER. If this process is blocked, the apoptotic drivers of the UPR are engaged while the cytoprotective mechanisms are suppressed [78]. Bortezomib is one such blocker; it is an inhibitor of the 26S proteasome's catalytic activity, and is clinically approved for the treatment of multiple myeloma, the ‘poster cancer’ for the UPR in tumors. There have been a number of clinical trials involving bortezomib for treatment of CNS tumors, but currently none are recruiting [63].

Our recent metabolomic work showed that recurrent GBMs produce more lipids of almost every category compared with primary GBMs, and those recurrent tumors have high lipogenesis enzyme expression [12]. As there are known connections between lipogenesis and the UPR (Figure 2), particularly via the IRE1/XBP-1 axis [79], and that link extends to FASN [80], combination therapies that disrupt long chain fatty acid synthesis in conjunction with targets involved in the UPR may have value in future GBM treatment strategies.

## Conclusion & future perspective

The stressful tumor environment drives stress responses in tumors that allow for their adaptation to this environment. The UPR is one such response that appears to benefit the ‘stressed’ tumor by enabling extended protein folding capabilities with high chaperone content, enhanced lipid biosynthesis for extracellular remodeling and generating metabolic profiles consistent with drug resistance [81] to deter treatment regimens. One could argue that GBMs adjust to the situation by making stress the ‘new normal’, benefitting from the cytoprotective aspects of the URP, but not suffering any of the consequences (i.e., apoptosis). While it is tempting to believe that application of additional stressors may ‘break’ the tumor, our attempts to do so clinically (e.g., with bortezomib in the treatment of multiple myeloma) lead nonetheless to even more resistant cells. The capacity for tremendous stress responses is a disturbing reality for most tumors, and GBMs display exaggerated hallmarks of stress resistance and antiapoptotic survival mechanisms. Perhaps our therapeutic efforts should focus on shaking the foundations of those stress responses, in combination with attacks on the ‘supply lines’, for example, the metabolism that feeds the tumors’ proliferative and invasive appetites.

**Executive summary**

The unfolded protein response (UPR) is an ancient endoplasmic reticulum (ER)-based cytoprotective mechanism evolved to protect cells from pathologic protein aggregation. It engages three arms to halt incoming protein load into the ER, to aid in protein folding of the molecules in the ER, to increase shipments out of the ER, to degrade excess unfolded proteins, and to initiate cell death if the stress is intractable.
Tumor cells, especially those from high grade gliomas/glioblastoma multiforme (GBMs), engage the UPR in various ways, perhaps initially as a stress response, but often seemingly to benefit from the increased outputs of proteins and lipids, without suffering the apoptotic consequences.
The high chaperone protein content of GBMs (and other brain tumors) implies that the folding pathways and the antiapoptotic pathways involving chaperones are critical features of such tumor biology.
These features, as well as those intertwined with the UPR, appear as provocative targets for drug intervention. However, to date few, if any, of those pharmacologics have had any significant impact on treatment regimens for patients with GBMs. We need further refinements in our understanding of tumor stress biology and its consequences for tumor survival and progression to appropriately design effective treatment strategies.
